# Supplementary material for: Machine learning classification of trajectories from molecular dynamics simulations of chromosome segregation
Source: PLoS One. 2022 Jan 21;17(1):e0262177. doi: 10.1371/journal.pone.0262177 (PMC8782305; doi:10.1371/journal.pone.0262177)
Supplement: S1 Appendix — (PDF) [file pone.0262177.s001.pdf]

S1 Appendix. Model for DNA. The length of bacterial chromosomes is about three orders of magnitude larger than the cell [77] [1]. Thus, cells have to massively compact their DNA in a manner that is compatible with DNA replication, DNA repair and further cellular processes [1]. Eukaryotes use histone proteins around which DNA is wrapped to form nucleosomes. Bacteria deploy a combination of different mechanisms to achieve this task [3]. Among the most prominent ones are entropic forces as a result of macromolecular crowding [33, 36–38, 78] and the partition of long DNA molecules into so-called supercoiled domains with fluid transitions between domain positions [77, 79–81]. It was found that the chromosome may be divided into 12 to 80 large domains having a size in the range of 25 kb to 100 kb, or into many more domains of around 10 kb on average [4]. Another mechanism used to achieve compaction is the association of DNA with nucleoid-associated proteins (NAPs). For example in *E. coli* the histone-like proteins HU, H-NS, FIS, and IHF play overlapping functions in chromosome compaction and bind to DNA with a preference for AT-rich sequences [3, 47]. In *B. subtilis* a similar protein, HBsu is known to play an important role for compaction [47]. While there is no direct evidence for the involvement of specific architectural proteins in chromosomal interaction domain (CID) boundary formation in bacteria, the involvement of the NAPs FIS and H-NS has been suggested for microdomain formation in *E. coli* [82]. The combination of these mechanisms leads to the organization of the chromosome into various domains on different length scales [1, 77, 83, 84].

In our simulations we used the bead-spring model of the chromosome [14, 85]. Here, the chromosome is modeled as a semi-flexible polymer which locally has the shape of a sphere (called bead) consisting of compacted DNA, due to the interactions with compaction proteins and negative DNA supercoiling. Thus, beads might be understood as topological domains. A bead typically contains one loop of DNA with a given loop size $L_{B,bp}$. With the length of one base pair of DNA $b = 0.34nm$ [26] we can calculate the bead radius as the radius of gyration

$$r_g = \frac{\sqrt{L_{B,bp}}b}{\sqrt{6}}. \tag{20}$$

Typically, we divide a chromosome of 4Mb into 80 domains of 50kb, which is in the range of the above mentioned domain sizes. Furthermore, the rather small number of particles is necessary to be able to perform a large number of MD simulations. The reason for this is the fact that the time scale of entropic segregation of confined ring polymers is dominated by the so called "induction phase" [14]. The induction phase is due to the fact that before segregation can start, the initial system symmetry has to be broken by spontaneous fluctuations. Because the length of the induction phase scales

exponentially with the length of the polymers [14], large numbers of beads per chromosome lead to an enormous computational cost in the MD simulations. The choice of 80 beads per chromosome is therefore a compromise between exact representation of chromosome details and numerical efficiency.
