# Supplementary material for: Machine learning classification of trajectories from molecular dynamics simulations of chromosome segregation
Source: PLoS One. 2022 Jan 21;17(1):e0262177. doi: 10.1371/journal.pone.0262177 (PMC8782305; doi:10.1371/journal.pone.0262177)
Supplement: S2 Appendix — (PDF) [file pone.0262177.s002.pdf]

S2 Appendix. Simulation setup. The bacterial cell is implemented as a constraint in the shape of a cigar. To simulate interactions between the chromosome and the membrane, we used a purely repulsive Weeks-Chandler-Anderson (WCA) potential. It is defined as

$$V_{WCA} = \begin{cases} 4\epsilon \left[\left(\frac{\sigma}{r}\right)^{12} - \left(\frac{\sigma}{r}\right)^{6} + c_{shift} \right] \text{ if } r_{min} < r < r_{cut} \\ 0 \text{ otherwise} \end{cases} \quad (21)$$

Here, we set $\sigma = d_B$ as the diameter of a bead, $c_{shift} = 0.25$ and $r_{cut} = 2^{\frac{1}{6}}\sigma = 1.1225\sigma$ thereby turning the Lennard Jones potential into a WCA potential, which is purely repulsive. This ensure that the beads were repelled by the cell wall and thus remained within the cell.

The electrostatic Debye-Hueckel potential was used to simulate the entropic repulsion between the beads. It is defined as

$$V_{DH} = \epsilon \frac{d_B}{r} \exp\left(-\frac{r}{d_B}\right), \quad (22)$$

for two particles of diameter d_B at a distance of r. The strength of the interaction is parameterized by ϵ. In our simulations we set $\epsilon = 1k_BT$ comparable to values used in similar studies [4, 14].

The beads of a chromosome are connected by elastic springs which are modeled by a harmonic potential

$$V_H(r) = \frac{1}{2}k(r - r_0)^2, \quad (23)$$

where we set $r_0 = 0$. Additionally, the spring constant k was adjusted so that the elastic energy compensates the repulsive Debye-Hueckel potential at extension $r_0 = d_B$ (i.e. in equilibrium).

To integrate the equations of motion `ESPResSO` uses the Velocity-Verlet MD integrator where we used a fixed time step of $t_{step} = 0.01\tau$. In order to keep the system at constant temperature we used a Langevin thermostat with a friction coefficient of $\gamma = \frac{5\epsilon m_B d_B}{\tau}$. With this choice we ensured that the motion was overdamped.
