# Supplementary material for: Machine learning classification of trajectories from molecular dynamics simulations of chromosome segregation
Source: PLoS One. 2022 Jan 21;17(1):e0262177. doi: 10.1371/journal.pone.0262177 (PMC8782305; doi:10.1371/journal.pone.0262177)
Supplement: S3 Appendix — (PDF) [file pone.0262177.s003.pdf]

S3 Appendix. Acceleration of MD time. With MD simulations the time-dependent dynamics of a molecular system is obtained by integrating Newton's equation of motion and sampling conformational space [49]. The time scale of the MD simulation results from the choice of the basic units for length, mass and energy. In the software package `ESPResSO` used in this study, these units are not predefined but must be selected by the user. All remaining units are derived from these choices [51]. In our simulations the particles are the beads of the chromosomes. Thus, the basic length scale is given by the diameter of a bead, $d_B \approx 30nm$, the basic mass scale is given by the mass of a bead $m_B \approx 1.08 \cdot 10^{-20} kg$ and as the basic energy scale we chose the thermal energy $\epsilon = k_B T \approx 4.14 \cdot 10^{-21} J$ at 300 K. With this we can calculate the basic time scale of our simulations, τ, as

$$[time] \quad = \tau = [length]\sqrt{\frac{[mass]}{[energy]}} \approx 4.8 \cdot 10^{-8} s. \tag{24}$$

Based on these numbers, it is clear that it is impossible to directly simulate of a process with duration of 20-60min. Instead, we need to find a way to speed up simulation time while accurately sampling conformational space. As already indicated in the main text, our simulation consists of a series of duplication events each leading to a new bead. After these duplication events, the replisomes continue to move along the chromosome until they reach the next beads to be duplicated. During this time, separation of the partially duplicated chromosomes takes place. The question at this point is how long it takes for the partially duplicated chromosomes to separate as much as possible. Since the daughter chromosomes are still connected to the parent chromosome (or the replication factory in the factory model), they cannot segregate completely. To estimate the time scale of mere separation, one can run simulations of two complete, initially overlapping chromosomes and determine after what time they are separated. We did this in our previous study [4] and found that pure entropic separation of chromosomes proceeds within a few μ s. Consequently, entropic segregation of chromosomes is a very efficient and fast process, so it can be assumed that the combined process of replication and segregation is dominated by replication time.

Another way to estimate the time it takes to completely sample conformational space after duplication of new beads is to calculate the ergodic measure. The ergodic measure was proposed by Thirumalai et al. [86] and Whitfield et al. [87] as an easily employed measure for the length of a simulation needed to adequately sample the thermodynamic important conformations at a given temperature. In other words, the ergodic measure is used to estimate the simulation length needed to guarantee self-averaging. For this, one calculates the mean-square difference between the average taken over a simulation α and the average taken over a simulation β, summed over all atoms of the system. The difference then provides a measure of the convergence of the two averages [86–88]. One way to define the ergodic measure is to consider the energies of the particles. In this case, the ergodic measure $\chi^2(t)$, can be defined as follows

$$\chi^2(t) = \frac{1}{N} \sum_{j=1}^{N} \left[\epsilon_{aj}(t) - \epsilon_{bj}(t)\right]^2 . \tag{25}$$

Here, $\epsilon_{aj}(t)$ is the time average energy for the j-th particle in simulation α and $\epsilon_{bj}(t)$ is the corresponding quantity for simulation β. N denotes the total number of particles. The test for ergodicity using $\chi^2(t)$ works as follows. If the system is ergodic at some point τ then $\chi^2(t)$ must vanish as the simulation time t approaches τ [86]. In S1 Fig the results of the calculation of the ergodic measure after duplication of a new bead in our simulations is shown. For this we computed the ergodic measure for 50 pairs of independent trajectories within our simulations. For every trajectory the total energy of the system was computed and used for the calculation of the entropic measure. The results of S1 Fig show that the entropic measure decays to zero at $\tau \approx 75\mu s$. This confirms our results from [4] and indicates that after the duplication of two new beads the average properties of the system correspond to equilibrium averages after $\approx 75\mu m$. Thus, we can jump to the next duplication step after this time is simulated. Thereby, we obtain an accelerated MD framework enabling us to perform realistic simulations by simulating a series of duplication events and subsequent separation of the partially replicated chromosome.

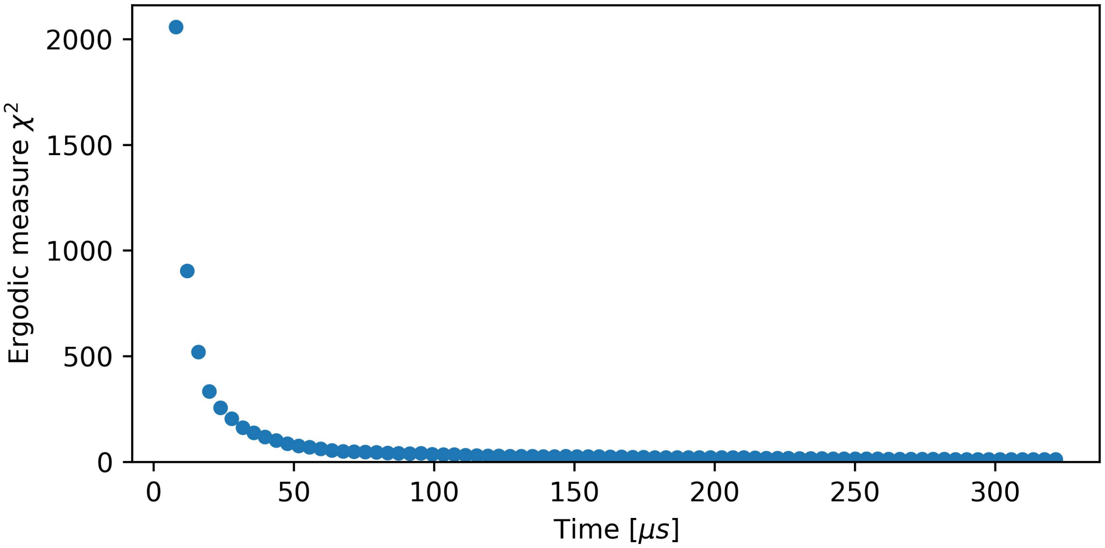

S1 Fig. Calculation of the ergodic measure after duplication of new beads in the simulations. For the calculation of the ergodic measure the total energies of the particles were calculated. The ergodic measure was calculated for 50 pairs of simulations and the results were averaged.
