# Supplementary material for: Machine learning classification of trajectories from molecular dynamics simulations of chromosome segregation
Source: PLoS One. 2022 Jan 21;17(1):e0262177. doi: 10.1371/journal.pone.0262177 (PMC8782305; doi:10.1371/journal.pone.0262177)
Supplement: S4 Appendix — (PDF) [file pone.0262177.s004.pdf]

S4 Appendix. Nested cross-validation.

The evaluation of the prediction accuracy of a ML model based on a single train/test split of the data might be biased due to a randomly favorable split of the data. Therefore, k-fold cross-validation is a commonly used statistical method to reduce this estimation bias. The idea is to randomly divide the data into k non-overlapping folds whereby one fold is used as a test set and the ML model is trained on the remaining $k-1$ folds. This procedure is repeated in a loop so that each of the k folds is used once as a test set. The final model performance is obtained by averaging the values computed in the loop [61]. Typically, one chooses $k = 5$ or $k = 10$ as values for the parameter k [58]. A further improvement of the cross-validation method is given by nested cross-validation. In this approach, hyperparameter tuning (see S5 Appendix) and evaluation of the model are combined by nesting two loops of cross-validation [55]. In the outer loop, the data is divided into five groups of training and test data. In the inner loop, each training set is then subjected to hyperparameter tuning, again using five-fold cross-validation. The model determined in this way is then trained on the full training data set of the outer loop and subsequently evaluated with the hold out test data. For the final result, the prediction accuracies of the five outer loops are averaged. The advantage of this method is that the probability of the hyperparameter search overfitting the data is reduced and a less biased estimate of the performance of a tuned model is obtained [55]. In our study we performed five-fold nested cross-validation for all our classifiers and summarized the results in S1 Table. The results of the nested cross-validation procedure confirm that the accuracies of the classifiers discussed in Table 3 and 5 are not based on a single randomly favorable split of the training and test data, but reflect the actual predictive ability of the classifiers. Since we train the classifiers on 80% of the data in each iteration as part of the five-fold cross-validation, S1 Table shows slightly better accuracies than for the simply evaluated classifiers trained on 70% of the data. Apart from this, the observations made earlier are confirmed, according to which the linear classifiers perform slightly better with the high-dimensional input vectors, while the ensemble classifiers are better suited for the approach based on low-dimensional input vectors. All classifiers achieve accuracies of more than 90% in the classification of trajectories, regardless of the choice of input vectors.

S1 Table. Prediction accuracies from nested cross-validation. Average prediction accuracies of the classifiers as obtained by five fold nested cross-validation.

**Model**	**Acurracy train set**	**Acurracy test set**
Random forest	0.999 (0.994)	0.931 (0.968)
Gradient Boosting	0.997 (0.998)	0.970 (0.974)
Logistic regression	0.985 (0.883)	0.973 (0.882)
SVM	0.999 (0.922)	0.992 (0.922)

Overall average prediction accuracies of the classifiers on the train and test data as obtained by five-fold nested cross-validation. The numbers before the brackets denote the results using high-dimensional input vectors while the numbers in the brackets denote the results for low-dimensional input vectors.
