# Supplementary material for: Machine learning classification of trajectories from molecular dynamics simulations of chromosome segregation
Source: PLoS One. 2022 Jan 21;17(1):e0262177. doi: 10.1371/journal.pone.0262177 (PMC8782305; doi:10.1371/journal.pone.0262177)
Supplement: S5 Appendix — (PDF) [file pone.0262177.s005.pdf]

S5 Appendix. Hyperparameter tuning. In order to find optimal model architectures for our classifiers we performed hyperparameter tuning. The set of hyperparameters define the architecture of a machine learning model and have to be defined before the actual learning process begins. The optimal hyperparameters for a

given problem are not known *a priori*. Therefore, one typically uses an automatic search in the space of possible hyperparamters to find the best possible setting. One possibility is offered by the `RandomizedSearchCV` function from the `scikit-learn` library by which a random search over possible hyperparameter settings can be performed. Thereby, our goal was to find hyperparameters yielding the best possible accuracy and avoiding overfitting at the same time. The hyperparameter tuning was performed exclusively on the training data to ensure that no information from the test data is learned by the models. The following hyperparameters have been optimized for the ensemble methods based on decision trees: The number of decision trees in the ensemble, the maximum depth of a tree (i.e. the maximum number of levels in each decision tree), the minimal number of samples required to split an internal node, the minimum number of samples required to be at a leaf node, the number of features to consider when looking for the best split (typical choices are the logarithm or the square root of the number of features) and a binary parameter to decide whether to use bootstrap samples when building trees or not (this parameter is only used for the random forest classifier) [20, 57, 61].

The hyperparameter tuning was performed two times for each classifier for both the high-dimensional and low-dimensional input vectors. In S2 Table and S3 Table the optimal hyperparameters for the tree-based classifiers using high-dimensional or low-dimensional input vectors are shown.

The hyperparameter tuning for the linear models was performed as a search over a grid of parameter values using the `GridSearchCV` library implemented in `scikit-learn` because the linear models have fewer hyperparameter to optimize. Both linear models use the parameter C to define the strength of the regularization. If a high value of C is chosen, regularization is low and one tries to fit the training data as well as possible. On the other hand, low values of C increase the generalization performance of the models and avoid overfitting. Our grid search revealed $C = 100$ for the SVM and $C = 1$ for the logistic regression classifier if high-dimensional input vectors were selected. For the low-dimensional input vectors we found $C = 100$ for the SVM and $C = 100$ for the logistic regression classifier. In addition to this setting, the following other parameters had to be defined: (i) for the SVM one has to define the kernel function, and (ii) for the logistic regression classifier a penalty function must be selected. For (i), we chose a linear kernel in order to be able to use the coefficients of the fitted classifier to compute feature importance values. This is only possible with a linear kernel where the fitted hyperplane and the coefficients are in the same dimensional space as the input vector of our features. For (ii), we selected the l_2 penalty function for the optimizer.

S2 Table. Optimized hyperparameters for tree-based classifiers using high-dimensional input vectors. Parameters for the tree-based classifiers trained on the complete trajectories. 50 different settings were tested with 3 rounds of cross-validation.

**parameter**	**Random forest**	**Gradient boosting**
Number of trees	800	700
Maximum depth of a single tree	20	10
Min. number of samples required to split an internal node	4	4
Min. number of samples required to be at a leaf node	10	6
Max. features	58	58
Bootstrap	True	-

Parameters for the tree-based classifiers trained on the high-dimensional input vectors. 50 different settings were tested with 3 rounds of cross-validation.

S3 Table. Optimized hyperparameters for tree-based classifiers using low-dimensional input vectors. Parameters for the tree-based classifiers trained on the complete trajectories. 50 different settings were tested with 3 rounds of cross-validation.

**parameter**	**Random forest**	**Gradient boosting**
Number of trees	700	700
Maximum depth of a single tree	90	70
Min. number of samples required to split an internal node	4	10
Min. number of samples required to be at a leaf node	12	4
Max. features	3	3
Bootstrap	True	-

Parameters for the tree-based classifiers trained on the low-dimensional input vectors. 50 different settings were tested wit 3 rounds of cross-validation.

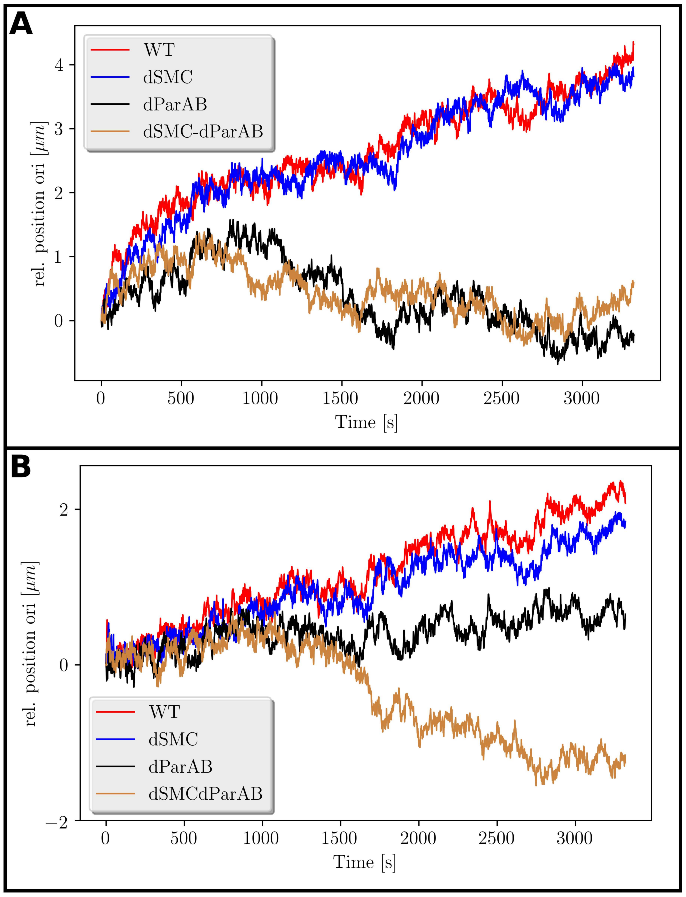

S2 Fig. Example trajectories of *ori* in the different cell types. A: Example trajectories of *ori* in the different segregation schemes for the track model of replication. The position along the long axis of the cell relative to the starting point is measured as a function of time. WT is shown in red, dSMC in blue, dParAB in black and dSMCdParAB in brown. **B:** Same as in A for the factory model of replication.

S4 Table. Average degree of separation after replication of both chromosomes. Average degree of separation of the two chromosomes after replication. Results are shown for the different cell types and averaged over 3000 runs per cell type. The degree of separation was defined as the longitudinal overlap of the chromosomes within the cell divided by the longitudinal elongation of the shorter chromosome.

	**Track model**	**Factory model**
WT	82.16	86.66
dSMC	83.00	91.54
dParAB	40.88	46.1
dSMCdParAB	66.64	42.10

Average degree of separation of the two chromosomes after replication. Results are shown for the different cell types and averaged over 3000 runs per cell type.

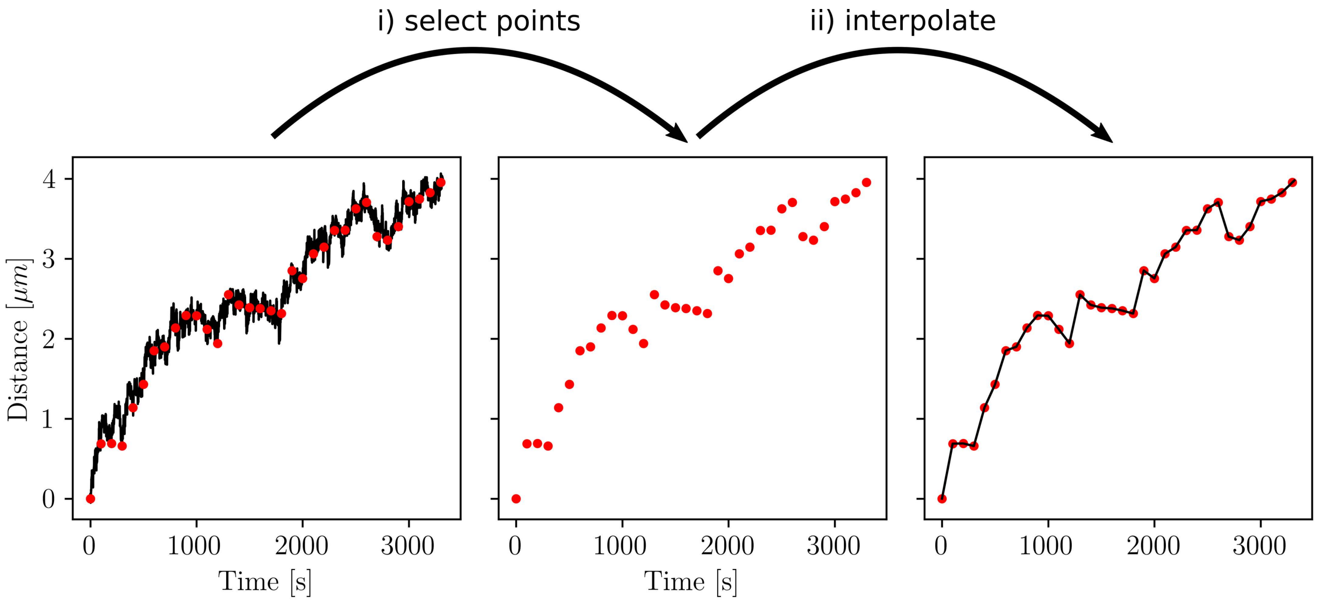

S3 Fig. Construction of trajectories with varying temporal resolutions. From the original trajectory shown in black in the left plot every 100-th point is selected to change the temporal resolution from the original 1s to 100s. In this way, an experiment with a reduced temporal resolution is mimicked. In the next step the selected points are interpolated to be able to compare trajectories of various temporal resolutions.
