# Supplementary material for: Machine learning classification of trajectories from molecular dynamics simulations of chromosome segregation
Source: PLoS One. 2022 Jan 21;17(1):e0262177. doi: 10.1371/journal.pone.0262177 (PMC8782305; doi:10.1371/journal.pone.0262177)
Supplement: Attachment — Submitted filename: Response to Reviewers.docx [file pone.0262177.s007.docx]

| Philipps-Universität \| Department of Physics \| 35032 Marburg  Editorial Office  PLOS One 1265 Battery Street, Suite 200 San Francisco, CA 94111 United States | **Department of Physics**  Prof. Dr. Peter Lenz  Phone: +49 6421 282 4326  Fax: +49 6421 282 4291  Email: [Peter.Lenz@physik.uni-marburg.de](mailto:Peter.Lenz@physik.uni-marburg.de)  Office: Mrs. Beatrix Payer  Phone: +49 6421 282 4201  Email: [Beatrix.Payer@physik.uni-marburg.de](mailto:Beatrix.Payer@physik.uni-marburg.de)  Address: Renthof 6, 35032 Marburg |
| --- | --- |
|  | Marburg, November 30, 2021 |

Dear Dr. Kestler,

We would like to thank you for the thorough evaluation of our manuscript “Machine Learning Classification of Trajectories from Molecular Dynamics Simulations of Chromosome Segregation” (Submission ID PONE-D-21-29250). We also thank the reviewers for their re-reading of our manuscript and the final helpful comment to improve our figure. We have addressed this last comment in the revised manuscript and described the changes we made in the detailed point-by-point response to the reviewers.

Sincerely yours and on behalf of all authors,

Peter Lenz

Point-by-point responses to reviewers' comments:

**Reviewer 1**

*General comments:*

*The authors have addressed adequately and thoroughly all of my concerns raised in the previous round of review. I will therefore recommend this work for publication after a final comment on Figure 8, which has been added in this latest version of the paper. For a clear visualization of the results, I think it would be helpful for the authors to provide an enlarged view of the very first part of the graph, showing the "Accuracy" for the values of the parameter "s" approximately in the range (0, 125), with labels showing the values of "s" for each data point entered, as an extra figure or within the figure itself. From the text I could only see that the very first point is evaluated for s = 5, but I'd like to know what other values were evaluated for this analysis, and better visualize how the "Accuracy" scales as the value of "s" increases in this very interesting range.*

We thank the reviewer for the positive evaluation of our manuscript. As suggested, we added a subplot to Figure 8 which shows an enlarged view of the first part of the graph for a better visualization of the results. We have also made this clear in the caption of the figure on p. 20 between l. 716-717 by adding the following sentence:

“The subplot provides an enlarged view of the very first part of the graph with the length of the trajectories ranging from 5s to 100s.”

**Reviewer 2**

Reviewer 2 stated that all comments were addressed and had no further requests for improvement.
